# Assessing the feasibility of evaluating and delivering a physical activity intervention for pre-school children: a pilot randomised controlled trial

**DOI:** 10.1186/s40814-016-0052-4

**Published:** 2016-02-18

**Authors:** Sally E. Barber, Cath Jackson, Catherine Hewitt, Hannah R. Ainsworth, Hannah Buckley, Shaheen Akhtar, Daniel D. Bingham, Ash C. Routen, Carolyn Summerbell, Gerry Richardson, Helen J. Moore, Kate E. Pickett, Claire O’Malley, Shirley Brierley, John Wright

**Affiliations:** 1 Born in Bradford Cohort Study, BiB Project Office, Temple Bank House, Bradford Royal Infirmary, Bradford Institute for Health Research, Duckworth Lane, Bradford, BD9 6RJ UK; 2 Department of Health Sciences, University of York, York, UK; 3 National Centre for Sport and Exercise Medicine (NCSEM), School of Sport, Exercise and Health Sciences, Loughborough University, Loughborough, UK; 4 School of Medicine, Pharmacy and Health, Durham University Queens Campus, Stockton-on-Tees, Durham UK; 5 Centre for Health Economics, University of York, York, UK; 6 Public Health, City of Bradford Metropolitan District Council, Bradford, UK

**Keywords:** Physical activity intervention, Pre-school children, Pilot randomised controlled trial, Process evaluation, Deprivation, Ethnicity

## Abstract

**Background:**

Few evidence-based physical activity interventions for pre-school children are available. This two-armed pilot cluster randomised controlled trial aimed to evaluate the feasibility of conducting a full-scale trial and of delivering an outdoor physical activity intervention for pre-school children.

**Methods:**

School was the unit of randomisation, and follow-up occurred at 10 and 52 weeks. Trial feasibility was assessed by recruitment, retention and completion rates of primary (daily moderate-to-vigorous physical activity (MVPA)) and secondary (anthropometric, quality of life, self-efficacy) outcomes. Potential effectiveness was assessed for the primary outcome using a linear regression model comparing MVPA between trial arms adjusting for clustering by school. Feasibility of delivering the intervention was assessed by intervention fidelity and attendance. Semi-structured interviews with parents, intervention facilitators, and head teachers explored acceptability and capability to deliver the intervention as well as acceptability of the study design.

**Results:**

Recruitment rates were 37 % of schools (*n* = 10 schools) and 48 % of pre-school children (*n* = 164 children). Retention of children to the trial at 52 weeks was 83.5 %. Thirty-nine percent of children had valid primary outcome accelerometer data at baseline and 52 weeks. Response rates for secondary outcome measures ranged from 52 to 88 % at 10 weeks and 59 to 80 % at 52 weeks. The mean difference in daily MVPA between trial arms at 52 weeks was 0.4, 95 % CI 16.3 to 17.0; *p* = 0.96. Fidelity of intervention implementation was 81 %. Intervention attendance was higher (82 %) during the summer initiation phase compared to autumn/spring initiation (50 %). Parents, facilitators and head teachers found the intervention acceptable and beneficial.

**Conclusions:**

Recruitment and retention rates suggest a trial in this outdoor setting with this population was feasible but is weather sensitive. However, strategies to increase accelerometer wear-time would need to be implemented for reliable primary outcome data to be obtained. There was high implementation fidelity by facilitators, and the intervention was seen as acceptable and deliverable. However, attendance was low and preliminary data showed no evidence of intervention effectiveness. A revised intervention, building on the successful elements of this pilot alongside adapting implementation strategies to improve attendance, should therefore be considered.

**Trial registration:**

Trial registry name and number: Current Controlled Trials, ISRCTN54165860. Date of registration: 4 September 2012.

## Background

Regular physical activity has important health and social implications for pre-school children including the promotion of healthy weight [1–4], development of bone and muscle and motor skills, improved social competence [5, 6] and reduction of cardiovascular disease risk [6–8]. There are distinct inequalities in physical activity for ethnic minority groups [9], and since levels of physical activity track into adulthood [10, 11], this disparity may translate into lifelong inequalities.

In 2011, the UK’s Chief Medical Officer published the ‘Start Active, Stay Active’ report [12] highlighting the need for activities to promote movement in the early years and recommending investment in community-level programmes in settings such as school playgrounds. However, there are very few effective, evidence-based programmes available for commissioning and none to the authors’ knowledge in the UK. In 2013, a meta-analysis of physical activity interventions for pre-schoolers (mean age 4.1 years) was published [13]. This analysis showed a small-to-moderate short-term effect of intervention on total physical activity (Hedges *g* = 0.44, SD = 0.86) and a moderate short-term effect on moderate-to-vigorous physical activity (MVPA) (Hedges *g* = 0.51, SD = 0.88) [13]. A further systematic review of physical activity and obesity prevention interventions in ethnic minority groups reported that none have been conducted in South Asian pre-school populations [14]. Two recent interventions, one in Germany and one in Belgium, have reported small increases in physical activity levels or reductions in sedentary behaviours which have been sustained over a longer term (6–12 months) [15, 16]. These results are promising; however, they may not be clinically meaningful given that the changes were small (less than 10 min difference in MVPA). Furthermore, the intervention in Belgium only reported a positive effect of the intervention upon boys and children of a high socioeconomic status [16]. Therefore, the development of pre-school physical activity interventions which focus on increasing physical activity levels in groups particularly at risk of low levels including low socioeconomic status children and ethnic minority groups is a pressing public health priority. In response, the pre-schoolers in the playground (PiP) intervention was developed. The process used to develop the intervention included focus groups with parents and consultation with early years workers. The process is described in detail elsewhere [17].

This pilot cluster randomised controlled trial (RCT) aimed to evaluate the feasibility of conducting a full-scale trial and of delivering an outdoor physical activity intervention for pre-school children, ‘PiP’, in a deprived multi-ethnic population. Potential cost-effectiveness was also assessed (reported elsewhere; [18]). The specific objectives were to determine (1) trial feasibility (recruitment, retention, feasibility of collecting outcome measures, preliminary assessment of intervention effectiveness) and (2) intervention feasibility (intervention fidelity, attendance, acceptability to parents, facilitators and head teachers and capability of the school to deliver the intervention).

## Methods

### Design

**Fig. 1:**
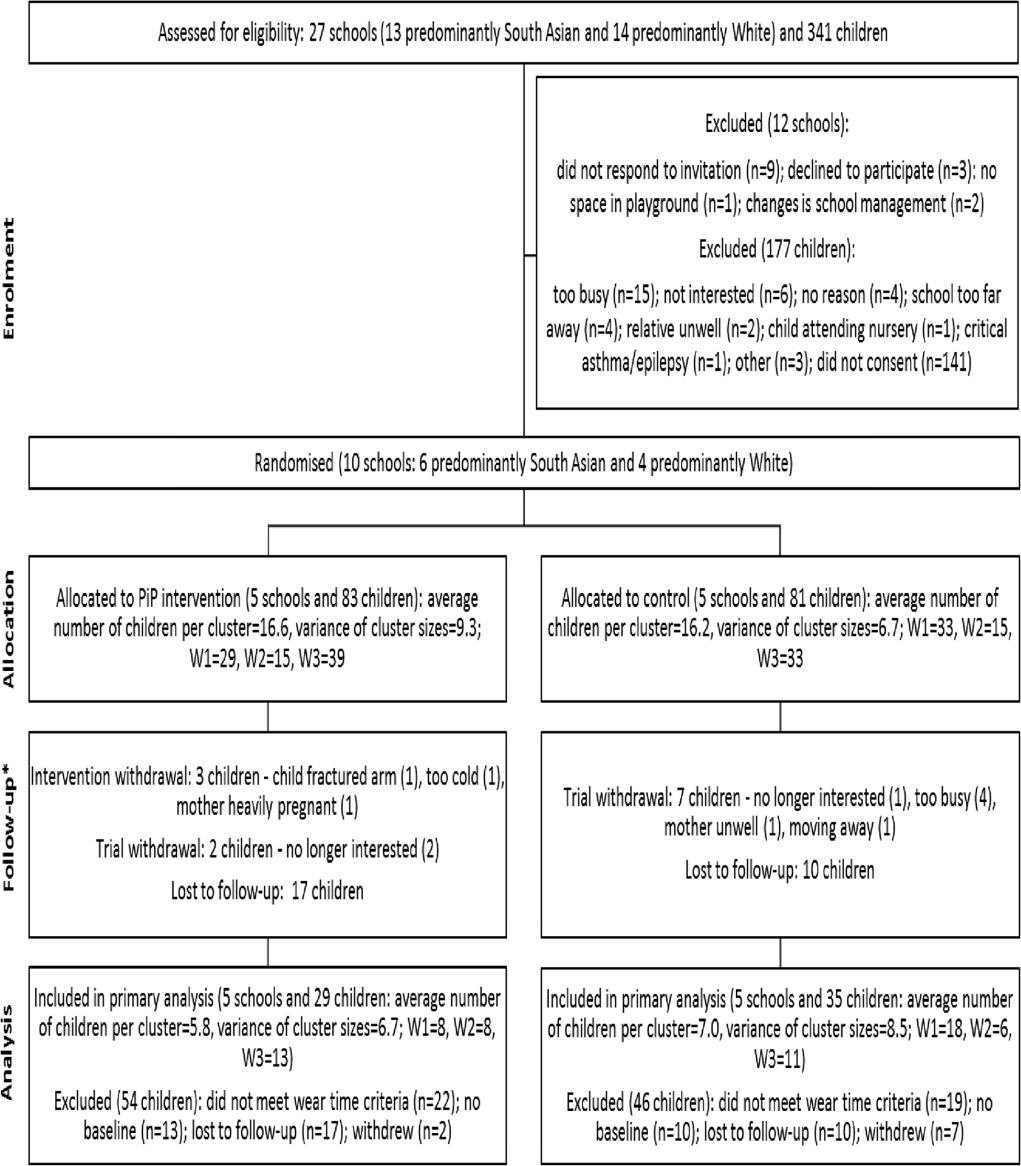
Cluster CONSORT diagram. *Based on primary outcome measure of accelerometer data irrespectively of meeting weartime criteria

The study was a two-armed pilot cluster RCT, comparing the PiP intervention and a usual practice (control) arm. The reporting of this trial follows the CONSORT statement recommendations [19]. The CONSORT checklist is provided as supplementary information, and the diagram is presented in Fig. 1. Recruitment, randomisation and implementation took place in three waves. Wave 1 commenced in the autumn of 2012, wave 2 in winter 2013 and wave 3 in summer 2013. The recruitment target was ten schools in the two poorest quintiles of index of multiple deprivation in the city of Bradford, UK, and 150 children aged 18 months to 4 years who were affiliated to these schools (attending feeder nurseries, children’s centres or with older siblings at the school). Recruitment was through letters sent home with school-going children attending the trial schools. Additionally, community research assistants recruited families through face-to-face conversations with parents or guardians in the playground at schools, children’s centres and nursery sites. To account for the linguistic diversity among the study population, research assistants recruiting families and subsequently conducting measurements and questionnaires were bilingual and undertook these tasks in either English or Urdu. The only exclusion criterion was if the parent or legal guardian was unable to provide consent. Schools were allocated on a 1:1 basis to either intervention or control. Randomisation was conducted by York Trials Unit randomisation service using a secure computer system after baseline data were collected. For wave 1, an even number of schools had to be allocated to the intervention and control, and so, block randomisation was used during this run in period. The subsequent six allocations (for waves 2 and 3) were achieved via minimisation to ensure balance of predominantly White and South Asian schools in the intervention and control arms. At least four clusters per arm are recommended for a cluster RCT to provide clear estimates of recruitment and follow-up [20]; the sample size thus exceeds recommendations for pilot trials [21]. Follow-up occurred at 10 and 52 weeks after the start of the intervention, finishing in May 2014. All outcomes were collected at each time point for participants in the intervention arm and the control arm. It was not possible for schools, participants and facilitators to be blind to allocation because of the nature of the intervention. It was planned that researchers would be blind to allocation; however, many of the parents informed staff of their child’s trial status. It was also planned that the statistician and health economist would be blind to allocation; however, due to staffing complications, an unblinded statistician conducted the statistical analyses.

### Consent and ethical approval

The study was ethically approved by the NRES committee Yorkshire and the Humber (12/YH/0334). Prior to participation, parents gave written informed consent for their own and their child’s engagement in the research. Head teachers and intervention facilitators also gave written informed consent for their participation in research interviews.

### PiP intervention arm

The intervention was delivered in primary school playgrounds at specific times to coincide with when school aged children were delivered to or collected from school, as identified by the school. Six 30-min PiP sessions per week were available for 30 weeks, and families were encouraged to attend three sessions a week. The initiation phase (10 weeks) was facilitated by a member of school staff. Each session included two 5-min structured-play activities for parents and children to engage in together (detailed in a manual), 15 min of free play (during which handouts were given and guided discussions conducted with parents) and an active tidy-up [17]. Children received a free piece of play equipment each week to take home and keep; the value of the whole play kit totalled £15. During the maintenance phase (20 weeks), playgrounds remained available six times a week for 30 min, but sessions were not supervised by school staff.

#### Control arm

Families in the control arm did not have access to a playground intervention and continued with their daily routines as normal.

### Trial feasibility

#### Recruitment and retention

Data relating to recruitment (number of schools and participants approached, excluded, agrees to further contact and consented) and retention (number of participants who withdrew, were lost to follow-up or who provided data) were captured using a central database. Parents and children attended measurement sessions either at participating schools or in their own homes at baseline, 10 weeks and 52 weeks.

#### Primary outcome

Parents were asked to place an ActiGraph GT3X+ accelerometer (ActiGraph Pensacola Florida, USA) on a waistbelt on their child (right anterior iliac crest) during waking hours for 7 days. Parents were asked to complete a wear-time log, detailing when the accelerometer was worn and removed; a reward sticker chart was given to the child to support them to wear the accelerometer. Raw count data was processed using ActiLife version 6 software (ActiGraph Pensacola Florida, USA) and integrated to 15 s epochs. Non-wear was considered to be consecutive zero counts of ≥10 min. A valid wear-time was considered to be any 3 days with ≥6 h of wear. Pate cut points [22] were used to estimate daily moderate-to-vigorous physical activity (MVPA; primary outcome), total and light physical activity, and sedentary time.

#### Secondary outcomes

Children’s height and weight were measured in lightweight clothing using a Leicester height measure (Harlow Healthcare, UK) and Seca electronic scales (Medical scales and measuring systems, UK). Body mass index (BMI) was calculated and converted to age- and sex-adjusted *z*-scores relative to WHO 2006 [23] growth standards using the least-mean-squared method. Upper arm circumference was measured at the midpoint between the acromion process and olecranon process. Waist circumference was measured at the midpoint between the lowest rib and iliac crest.

Parents completed the pediatric quality of life scales (infant or toddler scale depending on age of child) [24], the EQ5D-3L (parent quality of life measure) [25], ComQol-A5 [26] (parent well-being measure) and the general self-efficacy scale [27]. Parents also reported their child’s health and social care use (details of this are provided elsewhere; [18]). All questionnaires were completed electronically on tablet computers to minimise missing data or multiple answers. Families received a £10 gift voucher for attending measurement sessions.

### Intervention feasibility

#### Fidelity

Intervention implementation fidelity was assessed according to NIH Behavior Change Consortium guidance [28]; eight intervention sessions (≥1 at each school) were observed and fidelity scores relating to five key intervention factors (refer to Table 5) recorded.

#### Attendance to sessions and intervention harms

Participant attendance at intervention sessions was recorded at each session by the facilitator. Any accidents or injuries resulting from the intervention were to be recorded by the facilitator.

### Trial and intervention acceptability

Semi-structured interviews (a mix of face-to-face and telephone) were conducted with 15 parents from both trial arms (*n* = 10 intervention, *n* = 5 control), seven PiP facilitators and two head teachers from the intervention arm (see Table 1). For parents, a maximum variation sampling strategy [29] was employed to achieve diversity on three key characteristics: ethnicity, number of intervention sessions attended and parent (mother or father). All interviews were conducted in English and audio-recorded digitally. Parents were interviewed after completing their 10-week follow-up session and received a £10 gift voucher on completion of the interview. PiP facilitator interviews were also conducted at the 10-week follow-up, and head teachers were interviewed at the 52-week follow-up time point. Topics covered in the interview included acceptability of recruitment, study design and using the accelerometers (parents); views about intervention attendance (all) and intervention content (parents and facilitators); and schools’ capability and capacity to deliver the intervention (facilitators and head teachers). Interviews with parents lasted between 6 and 19 min. Facilitator interviews lasted between 13 and 31 min, and head teachers’ interviews lasted between 12 and 16 min.

**Table 1:** Characteristics of interview participants

		Intervention	Control	All
Parents		10	5	15
Wave	1	3	2	5
	2	5	3	8
	3	2	0	2
Parental role	Mother	9	4	13
	Father	1	1	2
Ethnicity	White	8	3	11
	South Asian	2	2	4
Self-reported attendance to PiP sessions	No sessions	4	N/A	4
	Less than 5 sessions	4	N/A	4
	Most sessions	2	N/A	2
PiP facilitators		7	N/A	7
Wave	1	3	N/A	3
	2	2	N/A	2
	3	2	N/A	2
Ethnicity profile of school	Predominantly White	2	N/A	2
	Predominantly South Asian	5	N/A	5
Head teachers		2	N/A	7
Wave	1	1	N/A	1
	2	0	N/A	0
	3	1	N/A	1
Ethnicity profile of school	Predominantly White	1	N/A	1
	Predominantly South Asian	1	N/A	1

### Statistical analysis

As this was a pilot trial, the analyses were mainly descriptive. For both trial arms, the numbers of schools and children approached, randomly assigned, receiving PiP or control, attending intervention sessions (intervention arm only) and providing outcome data for both the primary and secondary outcomes were summarised. Baseline, 10-week and 52-week follow-up data for primary and secondary outcomes were also summarised.

An analysis of the primary outcome to mimic practice in a full-scale trial was undertaken in Stata v12 using the intention-to-treat principle. School was the unit of analysis and children’s mean MVPA/day, the outcome variable. A weighted linear regression model compared the two arms weighted by the number of participants followed up in each cluster and adjusted for the baseline average MVPA/day for each cluster. A cost-effectiveness analysis was also conducted, and this is reported elsewhere [18].

### Qualitative data analysis

Audio recordings were transcribed verbatim with anonymisation of all personal data. These interview data were then analysed using thematic analysis which is a useful approach for producing qualitative analyses suited to informing the policy and programme development [30]. The six phases of thematic analysis were followed (familiarisation, generating initial codes, searching for themes, reviewing themes, defining and naming themes and producing the report [30]). The three data sets (parents, facilitators and head teachers) were analysed independently. The Atlas-ti software package facilitated data management.

## Results

### Trial feasibility

#### Recruitment and retention

Figure 1 shows participant flow through the trial. Twenty-seven schools were approached to take part in the study. Ten (37 %) consented and were randomised to the intervention or control arms (six with predominantly South Asian pupils and four with predominantly White pupils). Three hundred and forty-one children were screened for inclusion in the study, and no children were excluded from participating based on parent/guardian incapacity to consent. The parents of 305 (89 %) of these children agreed to be contacted further about the study, and 164 (48 %) children were ultimately consented to take part in the study. Nine (5.5 %) children withdrew from the trial, 27 (16.5 %) were lost to follow-up (providing no accelerometer data) and 137 (83.5 %) were retained in the trial (providing any data) at 52 weeks.

Demographics of the 164 trial participants are shown in Table 2. The average age of the children recruited was 2.8 ± 0.7 years. The number of boys and girls was similar (77 and 85, respectively), and there were more children of South Asian ethnicity than children of White ethnicity recruited into the trial (93 and 60, respectively). At baseline, mean MVPA for all children (intervention and control) was 63.6 ± 25.0 min per day and mean total PA was 285.0 ± 54.7 min per day. Ninety-six per cent of children met the guidelines of 180 min of total physical activity a day, and 35.7 % were considered overweight according to their BMI *z*-score. Randomisation achieved balance across age, sex and parental ethnicity. Attrition did not impact on the balance achieved at baseline in the ‘as analysed’ sample (i.e. those included in the primary analysis—those providing both baseline and 52-week MVPA data regardless of fidelity).

**Table 2:** Demographic and baseline characteristics

Characteristic	As randomised			As analysed		
	Intervention (*n* = 83)	Control (*n* = 81)	Total (*n* = 164)	Intervention (*n* = 29)	Control (*n* = 35)	Total (*n* = 64)
Age years, mean (SD)						
Child	2.7 (0.7)	2.9 (0.7)	2.8 (0.7)	2.8 (0.7)	2.8 (0.8)	2.8 (0.8)
Parent	29.4 (5.3)	31.7 (5.6)	30.6 (5.6)	30.9 (5.9)	33.2 (5.4)	32.2 (5.7)
Sex, *n* (%)						
Child						
Female	43 (52.4)	42 (52.5)	85 (52.5)	14 (50.0)	17 (50.0)	31 (50.0)
Male	39 (47.6)	38 (47.5)	77 (47.5)	14 (50.0)	17 (50.0)	31 (50.0)
Parent						
Mother	80 (96.4)	77 (95.1)	157 (95.7)	28 (96.6)	32 (91.4)	60 (93.8)
Father	3 (3.6)	4 (4.9)	7 (4.3)	1 (3.5)	3 (8.6)	4 (6.3)
Ethnicity, *n* (%)						
Child						
White	35 (42.7)	25 (30.9)	60 (36.8)	9 (31.3)	9 (25.7)	18 (28.1)
South Asian	43 (52.4)	50 (61.7)	93 (57.1)	19 (65.5)	25 (71.4)	44 (68.8)
Other^a^	4 (4.9)	6 (7.4)	10 (6.1)	1 (3.5)	1 (2.9)	2 (3.1)
Parent						
White	34 (44.2)	28 (43.1)	62 (43.7)	9 (33.3)	9 (33.3)	18 (33.3)
South Asian	41 (53.3)	36 (55.4)	77 (54.2)	17 (63.0)	18 (66.7)	35 (64.8)
Other^a^	2 (2.6)	1 (1.5)	3 (2.1)	1 (3.7)	0 (0.0)	1 (1.9)
Physical activity	*n* = 55	*n* = 58	*n* = 113	*n* = 29	*n* = 35	*n* = 64
Minutes spent in MVPA/day, mean (SD)	64.2 (26.3)	62.0 (22.9)	63.1 (24.5)	65.6 (26.7)	61.9 (23.7)	63.6 (25.0)
Minutes spent in light activity/day, mean (SD)	212.8 (44.1)	212.6 (40.2)	212.7 (41.9)	224.1 (41.6)	219.2 (36.8)	221.4 (38.8)
Minutes spent sedentary/day, mean (SD)	317.7 (89.1)	287.4 (64.8)	302.2 (78.7)	342.8 (89.3)	296.8 (61.5)	317.7 (78.3)
Minutes of total PA/day, mean (SD)	277.0 (60.2)	274.6 (56.0)	275.8 (57.8)	289.7 (57.2)	281.1 (53.1)	285.0 (54.7)
Percentage of children ≥180 min/day (%)	53/55 (96.4)	55/58 (94.8)	108/113 (95.6)	29/29 (100.0)	33/35 (94.3)	62/64 (96.9)
Anthropometry						
Height in centimetres, mean (SD)	*n* = 72	*n* = 73	*n* = 145	*n* = 26	*n* = 30	*n* = 56
	92.4 (7.3)	93.1 (6.9)	92.8 (7.1)	93.5 (8.2)	92.8 (7.8)	93.1 (7.9)
Weight in kilograms, mean (SD)	*n* = 74	*n* = 77	*n* = 151	*n* = 28	*n* = 34	*n* = 62
	14.1 (2.5)	14.3 (2.3)	14.2 (2.4)	14.4 (2.6)	14.3 (2.6)	14.3 (2.6)
Abdominal circumference in centimetres, mean (SD)	*n* = 60	*n* = 66	*n* = 126	*n* = 22	*n* = 27	*n* = 49
	50.0 (3.8)	50.2 (3.9)	50.0 (3.8)	49.6 (3.4)	50.6 (4.3)	50.1 (3.9)
Arm circumference in centimetres, mean (SD)	*n* = 55	*n* = 66	*n* = 121	*n* = 21	*n* = 27	*n* = 48
	16.9 (1.8)	17.3 (3.9)	17.1 (3.1)	17.0 (1.8)	18.3 (5.9)	17.7 (4.6)
BMI *z*-score, mean (SD)	*n* = 71	*n* = 73	*n* = 144	*n* = 26	*n* = 30	*n* = 56
	0.6 (1.1)	0.8 (1.1)	0.7 (1.1)	0.5 (1.1)	1.0 (1.1)	0.7 (1.1)
Overweight, BMI *z*-score ≥1.04, *n* (%)	*n* = 71	*n* = 73	*n* = 144	*n* = 26	*n* = 30	*n* = 56
	24 (33.8)	29 (39.7)	53 (36.8)	8 (30.8)	12 (40.0)	20 (35.7)
Well-being	*n* = 75	*n* = 72	*n* = 147	*n* = 27	*n* = 30	*n* = 57
ComQol-A5 total objective score, mean (SD)	73.2 (8.0)	75.2 (7.1)	74.2 (7.6)	74.7 (7.6)	76.3 (6.9)	75.6 (7.2)
Self-efficacy	*n* = 83	*n* = 80	*n* = 163	*n* = 29	*n* = 34	*n* = 63
General self-efficacy, mean (SD)	30.3 (5.7)	29.1 (5.4)	29.7 (5.6)	30.6 (4.6)	30.0 (5.3)	30.3 (4.9)

Data were missing for child gender (*n* = 2), child ethnicity (*n* = 1) and parent ethnicity (*n* = 22)

^a^African, Burmese and dual heritage (White, Asian, Chinese and Indian)

#### Primary outcome

At baseline, 113 (69 %) children returned valid accelerometer data. Sixty-four (39.0 %) children had MVPA data at baseline and 52-week follow-up and were included in the primary analysis.

Table 3 shows that the intervention arm appears to be undertaking slightly less MVPA, more light activity and be more sedentary compared to the control arm at 52-week follow-up. The minutes in total physical activity and the percentage of children undertaking 180 min of physical activity a day were higher in the intervention at 10 weeks but similar at 52 weeks. At 52 weeks, the adjusted mean daily MVPA was 75.6 (95 % CI 63.4 to 87.8) in the intervention and 75.2 (95 % CI 64.2 to 86.3) minutes in the control arm (mean difference 0.4, 95 % CI 16.3 to 17.0, *p* = 0.96).

**Table 3:** Summaries of physical activity at 10 and 52 weeks of follow-up

Physical activity domain	Intervention	Control	Total
Minutes spent in MVPA/day, mean (SD)			
10 weeks	61.8 (25.2)	62.1 (22.9)	61.9 (24.0)
52 weeks	72.6 (30.4)	74.3 (25.5)	73.5 (27.8)
Minutes spent in light activity/day, mean (SD)			
10 weeks	212.9 (39.4)	200.4 (45.1)	206.7 (42.5)
52 weeks	220.9 (37.4)	219.1 (43.1)	220.0 (40.3)
Minutes spent sedentary/day, mean (SD)			
10 weeks	318.0 (102.6)	303.7 (69.0)	310.9 (87.4)
52 weeks	335.7 (102.4)	302.5 (65.6)	318.5 (86.5)
Minutes of total PA/day, mean (SD)			
10 weeks	274.6 (49.3)	262.6 (61.1)	268.7 (55.5)
52 weeks	293.5 (52.0)	293.4 (59.3)	293.5 (55.0)
Children achieving 180 min/day (%)			
10 weeks	44/44 (100.0)	41/43 (95.4)	85/87 (97.7)
52 weeks	41/42 (97.6)	44/45 (97.8)	85/87 (97.7)

#### Secondary outcomes

Table 4 shows anthropometric outcomes alongside well-being and self-efficacy total scores. Response rates for secondary outcomes ranged from 52 to 88 % at 10 weeks and from 59 to 80 % at 52 weeks. There was little difference in average body mass and height between the intervention and control arms at either follow-up point. At 10 weeks, the average BMI *z*-value in the intervention arm was slightly lower at 0.5 (SD 1.2) than in the control arm which was 0.7 (SD 1.2); values at 52 weeks were more similar. The average waist and arm circumference were slightly lower in the intervention arm than in the control arm at both time points. In terms of well-being, the overall objective score was lower in the intervention arm at both time points [10 weeks: intervention 72.7 (SD 9.2), control 74.0 (SD 6.7); and 52 weeks: intervention 72.4 (SD 7.5), control 74.5 (SD 7.1)]. However, there was little change in scores between the two time points. Self-efficacy scores were similar between allocated arms at both time points [10 weeks: intervention 31.1 (SD 6.4), control 30.9 (SD 5.0); and 52 weeks: intervention 31.8 (SD 5.5), control 31.9 (SD 4.3)].

**Table 4:** Summaries of anthropometric, well-being and self-efficacy measures at 10 and 52 weeks of follow-up

Characteristic	Intervention (*n* = 83)	Control (*n* = 81)	Total (*n* = 164)
	*n*, mean (SD)	*n*, mean (SD)	*n*, mean (SD)
Body mass in kilograms			
10 weeks	58, 14.6 (2.6)	58, 14.6 (2.3)	116, 14.6 (2.4)
52 weeks	60, 16.1 (2.8)	58, 16.4 (2.8)	118, 16.2 (2.8)
Height in centimetres			
10 weeks	53, 95.0 (6.8)	57, 95.1 (6.4)	110, 95.1 (6.6)
52 weeks	58, 100.2 (7.2)	57, 100.8 (6.7)	115, 100.5 (6.9)
BMI *z*-score			
10 weeks	71, 0.5 (1.2)	73, 0.7 (1.2)	144, 0.6 (1.2)
52 weeks	54, 0.2 (1.2)	53, 0.3 (1.2)	107, 0.3 (1.2)
Waist circumference in centimetres			
10 weeks	47, 49.6 (3.7)	41, 50.4 (4.8)	88, 49.9 (4.2)
52 weeks	53, 51.1 (4.1)	50, 52.2 (4.8)	103, 51.6 (4.5)
Upper arm circumference in centimetres			
10 weeks	45, 17.0 (1.4)	41, 17.8 (5.5)	86, 17.4 (3.9)
52 weeks	50, 17.5 (2.1)	47, 17.6 (1.5)	97, 17.5 (1.8)
ComQol-A5 total objective score			
10 weeks	62, 72.7 (9.2)	53, 74.0 (6.7)	115, 73.3 (8.1)
52 weeks	65, 72.4 (7.5)	60, 74.5 (7.1)	125, 73.4 (7.4)
General self-efficacy score			
10 weeks	64, 31.1 (6.4)	66, 30.9 (5.0)	130, 31.0 (5.7)
52 weeks	68, 31.8 (5.5)	63, 31.9 (4.3)	131, 31.9 (5.0)

### Intervention feasibility

#### Fidelity

The mean total fidelity score was 29.1 (SD 5.7), out of a total possible score of 36, highlighting overall good adherence (81 %) to the intervention protocol. Two factors that had poorer adherence were ‘Encouraging families to seek other physical activities on non-intervention days’ and providing ‘Information on guidelines for physical activity for under 5’s’ (Table 5).

**Table 5:** Summary of fidelity scores across five key intervention factors

Fidelity component assessed	Mean (SD)	Median (min, max)
1. Delivery as per manual		
Welcome and 5 min structured play	3.69 (0.46)	4 (3, 4)
Encourage families to seek other PA	2.13 (1.09)	1.75 (1, 4)
Information on guidelines for PA for under 5’s given	2.00 (1.41)	1 (1, 4)
5 min structured play and goodbye	3.44 (1.05)	4 (1, 4)
Total score	11.25 (3.30)	10.25 (7, 15)
2. Supervision	3.56 (0.73)	4 (2, 4)
3. Support given to parents	3.31 (0.96)	4 (2, 4)
4. Encouragement of children	3.81 (0.37)	4 (3, 4)
5. Infusion of play equipment	3.56 (0.50)	3.75 (3, 4)
Total fidelity score	29.13 (5.74)	29.50 (21, 35)

#### Attendance to sessions and intervention harms

There were no accidents of or injuries related to the intervention reported by the PiP facilitators. Table 6 displays attendance at PiP sessions. Attendance was markedly higher during the summer-term initiation phase and greater among South Asian children (mean 11.3, SD 11.8; median 4.5, min 1 to max 35) compared to White children (mean 10.7, SD 12.1; median 3, min 1 to max 33). Attendance was also much higher in the initiation phase compared to the maintenance phase. It was recommended that children attend 30 sessions in the initiation and 60 in the maintenance phase (*n* = 90 overall). No schools delivered the second term of the maintenance phase, so the maximum number of sessions that families could have attended (initiation plus maintenance) was 60.

**Table 6:** Attendance of children to initiation and maintenance phases of the intervention according to school

Predominant ethnicity of intervention group	School 1: White	School 2: South Asian	School 3: South Asian	School 4: White	School 5: South Asian	Overall
Initiation	Autumn/winter	Autumn/winter	Winter/spring	Summer	Summer	
Number of children	13	16	15	18	21	83
Attended any session, *n* (%)	6 (45.2)	10 (62.5)	6 (40.0)	15 (83.3)	17 (81.0)	54 (65.1)
Number of sessions attended, mean (SD)	1.8 (1.0)	2.3 (1.4)	10.7 (10.6)	10.8 (10.7)	13.6 (9.8)	9.1 (9.6)
Number of sessions attended, median (min, max)	1.5 (1, 3)	2 (1, 5)	10 (1, 21)	5 (1, 28)	17 (1, 29)	3 (1, 29)
Maintenance	Winter/spring and summer	Winter/spring and summer	Summer and autumn	Autumn and winter/spring	Autumn and winter/spring	
Number of children	13	16	15	18	21	83
Attended any session, *n* (%)	0 (0.0)	0 (0.0)	3 (20.0)	4 (22.2)	6 (28.6)	13 (15.7)
Number of sessions attended, mean (SD)	0.0 (0.0)	0.0 (0.0)	8.3 (1.2)	3.0 (2.3)	5.0 (3.7)	5.2 (3.4)
Number of sessions attended, median (min, max)	0 (0, 0)	0 (0, 0)	9 (7, 9)	3 (1, 5)	4.5 (1, 11)	4.5 (1, 11)
Overall						
Number of children	13	16	15	18	21	83
Attended any session, *n* (%)	6 (46.2)	10 (62.5)	6 (40.0)	15 (83.3)	17 (81.0)	54 (65.1)
Number of sessions attended, mean (SD)	1.8 (1.0)	2.3 (1.4)	14.8 (15.2)	11.6 (12.1)	15.4 (11.6)	10.3 (11.5)
Number of sessions, median (min, max)	1.5 (1, 3)	2 (1, 5)	14.5 (1, 30)	5 (1, 33)	5 (1, 33)	3.0 (1, 35)

### Trial and intervention acceptability

#### Acceptability of recruitment and study design

In the interviews, parents were generally positive about being approached in the playground to take part in the study, and this appeared to be associated with the friendly manner of the community research assistants and the assumption that the school/nursery/children’s centre had endorsed the PiP study.

I don’t usually like being approached by people in town. This was OK as was in the school playground so you are more trusting.

P01—control, wave 1, father, White

Two parents for whom English was not their first language said they had been unable to read the study information sheet but that the community research assistant had explained the study to them verbally, which had helped with their understanding.

Yeah, because I am, sorry, but I’m tell her, I said my reading problem, I’m not properly read, but I said you some explain and I understand what she said.

P02—control wave 2, mother, Pakistani

Other parents commented that the study information was clear both from the conversation with the community research assistant and in the written information that they were given to take home.

Many of the parents, however, were confused about the concept of randomisation and its implication for their participation; for example one parent thought that the schools with the most children were chosen for the PiP intervention.

I thought the most school that had the most parents, that’s why they got chosen [for the PiP intervention].

P13—intervention, wave 2, mother, White, attended no PiP sessions

#### Acceptability of accelerometry

Most parents said that using the accelerometer belt was easy and generally described their child as ‘being fine’ or enjoying wearing the belt.

Yeah, he loves the belt… it’s easy when you put it on in the morning, but when you take it off in the night it’s, it’s hard because he says, oh I have to leave it on… but when he’s gone to sleep I just pull it off.

P13—intervention, wave 3, mother, Pakistani, attended most PiP sessions

Some parents mentioned difficulties in using the belt, namely they forgot to put it on the child, the belt either moved or pinched the child when tight and a small part of the accelerometer kept falling off (and could be swallowed by the child).

Quite often you’ve got to move it back into place, cause if she’s moving about and stuff, it’s moving. So it does need summat a bit more so it’ll stay in that spot. The other problem is it, it’s got small parts on it, as well, that come off.

P10—intervention, wave 2, father, White, attended one PiP session

#### Views about intervention attendance

A variety of reasons were offered by parents and facilitators for poor attendance to the PiP sessions. Some of these were family issues and unrelated to PiP, for example child and family illness. The timing and location of the PiP sessions were discussed by several parents as a barrier to attendance, particularly in the context of their children’s routines (nap and nursery times).

My daughter is in Nursery until 11.45 and then she needs to nap when gets home, so hard to get to afternoon session. This term I will ask Nursery if I can take her out 20 minutes in the morning to do the exercise, should be ok as it’s at the same place.

P05—intervention, wave 1, mother, White, attended a few PiP sessions

Whereas for the parents who attended regularly, the timings and location worked as anticipated, linking with school drop-off and pick-up. One of the facilitators described how one mum had ‘made it work’ by doing two sessions on one day each week to fit all three sessions in around her employment.

The bad weather was discussed by all seven parents in waves 1 and 2 (autumn and winter initiation phases) and offered as a reason for why ‘other parents and children’ may not have attended. Facilitators and head teachers also talked about the negative influence of the weather suggesting that it was to be expected that parents did not return when they had been outside in ‘freezing’ weather. A facilitator in wave 3 described the opposite problem of parents not bringing their children as it was too hot.

The attendance was a problem cause of the time of the year. The weather played a huge, a major factor in the parent, getting the parents to attend. Any adverse weather and attendance fell there. If a similar project was to be run again it would be better in the summer.

H01, wave 1, South Asian school

The facilitators offered some additional reasons why they thought the parents had not attended PiP sessions; these included a lack of promotion of the study at the school, the ‘burden’ of the measurement sessions, unfamiliarity with the facilitator, feeling embarrassed being watched doing the PiP sessions on the playground, the challenge of bringing two children to the sessions and a nervousness and reluctance by parents to take part in school activities. Both of the head teachers spoke about children who would most benefit from attending being the ones whose families are most difficult to engage in school activities generally.

Finally, two facilitators were not confident that the ‘maintenance’ sessions would work without them being there because they believed that someone needed to take the lead. A different two were more positive because they thought that there were some parents who were regular attenders who were very capable of running the sessions, but they were less sure that parents with less confidence would continue to attend.

And you know it’s on and you know it’s there, because they can see me out on the playground, cause most of them know me anyway from other things, and I think they just feel like there’s someone there to guide them really.

W04—wave 1, White school

#### Acceptability of intervention content

Five of the nine parents in the intervention arm who were interviewed had attended any PiP sessions, and two of these had attended most sessions (and provide the most detail about the sessions). The other three had attended less than five sessions. Four of the five parents and all of the facilitators were very positive about the PiP sessions. They saw them as fun for the children and commented on the variety of games and equipment that the children had enjoyed playing with both in the sessions and then at home. In contrast, one parent reported a negative experience of the intervention and only attended one session, explaining that the facilitator was not confident in delivering the session.

We have enjoyed ourselves. It’s like every week it was something different. Something different every day and my (name of child) loves playing with balls and the cricket bat.

P13—intervention, wave 3, mother, Pakistani, attended most PiP sessions

Because I’m dragging the equipment up before me, they do a little bit of free play before we begin even. They’ve normally started kicking the ball, they just do, they don’t in the beginning the first few weeks they’ve probably hung around and waiting for me to start, now as soon as there’s a ball or a hoop, they’re off basically.

F05—wave 3, White school

The two parents who had frequently attended the sessions identified how their children had developed over the 10 weeks, not only in initiating playing active games but also in developing confidence, learning colours, sharing toys with other children and making friends with children who would also be starting nursery in the following September.

When she started she was very clingy, you know she wouldn’t do anything, she wouldn’t move around, she just wanted to hold me and look, she was interested in what the other children were doing, she wasn’t doing it herself, but over the weeks, so I’ve noticed the change in her. So you know she’s picking the bean bag up, she loves the beanbag.

P14—intervention, wave 3, mother, Pakistani, attended most PiP sessions

Parents and facilitators also spoke about benefits to the parents of attending the sessions; these included making new friends, enjoying doing something with their child, being more active themselves and learning how to do active play with their child at home.

When we model it from the first session it’s getting to, used to it, and then eventually they (the parents) would know what to do. The more we did we had more fun, I think because we were getting more confident at delivering it and the parents were getting more confident with us as well, yeah and the kids.

F02b—wave 1, South Asian school

#### Capability and capacity to deliver the intervention

Both head teachers described how they had incorporated the work into the staff’s existing workloads. One viewed this as a cost to the school and commented that in order for the intervention to continue, external staff would be required to deliver the sessions.

We provided a couple of members of staff to keep the project running… It did take ‘em away from their normal job… On the whole, the project did impact on children and staffing at (name of school). (The research team) would need to provide more staff in the future should the project run again.

H01—wave 1, South Asian school

The other described how the school was well staffed with a good budget so this was less of a concern. However, she explained that parents at her school do not ‘stick with’ any initiatives for very long, so a shorter programme may be more sustainable.

## Discussion

Recruitment of schools to the trial (10/27, 37 % uptake) was similar to another school-based physical activity intervention (41 % uptake) [13], and recruitment of families (164/341, 48 % uptake) was marginally better than other similar studies (39–42 % uptake) [16, 31, 32]. Retention was also better than other similar trials with 83.5 % at 52 weeks compared to 68 and 75 % at 12 weeks [33]. At baseline, 113 (69 %) children returned valid accelerometer data; however, those who provided valid data at both baseline and 52 weeks was lower (*n* = 64, 39 %). This aligns with similar RCTs in older pre-school children (mean age ~5 years), where 42 to 70 % of children returned valid accelerometer data at both baseline and follow-up [15, 16, 34]. The interview data from parents suggested that the recruitment strategy was acceptable; however, parents did not understand the concept of randomisation. Accompanying written information in the participant information sheet with a visual description pictorially or via an online video may help parents to better grasp this concept. The accelerometers were acceptable for most children. To enhance the safety of using the accelerometers with very young children, we suggest encapsulating the monitors in a waterproof adhesive dressing (such as Tegaderm™) to prevent small parts being accessed. Ways to improve wear-time could include improving communication to parents that the belts can be worn over clothing to prevent pinching and providing daily morning telephone calls/text messages to parents, reminding them to fit the belts to their children.

Attendance was greater during the summer initiation phase when the weather was dryer and warmer. The interview data showed clearly that the weather and associated child sickness in the colder months impacted on attendance. Parents, facilitators and head teachers all recommended a summer-term only intervention. Attendance was also poor during the maintenance phase, with facilitators suggesting this may be due to the lack of their presence in the playground. Indeed, previous research has shown that teacher-led interventions are more effective than parent-led interventions for pre-school children [13]. Furthermore, one head teacher said that with the maintenance phase the intervention became too long for parents to engage with. Based on the interview data, several modifications to the intervention and strategies to maximise attendance are proposed. These are as follows: a shorter, 10-week (one term) summer-time-only intervention, delivered by a facilitator, with more visible promotional materials, reminders to attend and incentives for parents (healthy refreshments and social time).

Despite the low attendance rates overall, parents, facilitators and head teachers all commented that the intervention was beneficial to children, parents and schools. These benefits were not directly related to physical health, rather, learning, developing social and communication skills and building up relationships between families and schools. Gordon et al.’s [13] recent meta-analysis of physical activity interventions for pre-school children emphasised the importance of the delivery site and showed that effective interventions were delivered in a learning environment (usually a pre-school setting), whereas home-based interventions were associated with a small negative effect upon indices of physical activity. This highlights the importance of using school/pre-school sites for health promotion. Emphasising the benefits that health interventions may have to learning and learning environments may increase the willingness of these settings to engage with and deliver health interventions.

Previous pre-school physical activity trials rarely report on the fidelity of the intervention. One teacher-led pre-school structured-play physical activity intervention from the USA [35] reported an average of 70 % adherence across different domains included in their analysis (duration of sessions and delivery according to instructions). In the current study, fidelity of the intervention was good indicating that it was feasible for schools to deliver the intervention. The two components with poorer adherence in the PiP intervention were ‘providing information on guidelines for physical activities for the under 5’s’ and ‘encouraging families to seek other physical activities’. These two components should have been covered in the guided discussion and handouts section of the PiP sessions which parents recalled little information about. Changing the way that this information is delivered, perhaps via short information video clips, may be one way to meet these intervention objectives.

For the qualitative aspect of the study, there was good representation from parents and from the school staff who were delivering the intervention. Head teachers were more difficult to engage in the interviews with only two from the five intervention schools taking part. In future work, we suggest that head teachers delegate liaison with the PiP research team to a member of their senior staff with particular responsibility for physical activity, for example the PE coordinator. The two schools where the head teachers were interviewed had different views on the capacity of their schools to deliver the intervention. Both had incorporated the delivery of the intervention into the workloads of existing staff. In one school, the facilitators were nursery staff, and in the other school, the facilitator was a parental involvement worker whose role is to work with and support families. This seemed to be a more appropriate member of staff to facilitate the intervention as it was more easily incorporated into their workload. In future, these members of staff would be sought to deliver the intervention. In addition, leverage can be sought with the new English schools inspection monitoring criteria (Ofsted) coming into force in all school levels (including early years settings) in September 2015, which will include a new judgement criterion focus on school promotion of physical activity and physical activity-related policies [36].

The current study has both strengths and limitations. The pilot trial used a multi-method approach (quantitative, qualitative and health economic analyses); thus, a thorough evaluation of the feasibility of both conducting a trial and delivering the PiP intervention has been conducted. The study was successful at engaging hard-to-reach White and ethnic minority families living in areas of high deprivation, groups which are often underrepresented in research. There were good recruitment and retention rates. However, a relatively low number of participants were included in the analysis of data because few had accelerometry data at baseline and 52-week follow-up. Where families are living with financial pressures, putting an accelerometer on their child may not be a priority. This is a limitation of the pilot trial, and interview data from the current study offers potential solutions to increase wear-time. There were poorer-than-expected attendance rates to the intervention, and consequently, the data relating to the effect of the intervention on health outcomes may not reflect the likely effect of the proposed modified intervention.

## Conclusions

Recruitment and retention rates suggesting a full-scale trial in this outdoor setting with this population would be feasible. In order for reliable primary outcome data (habitual physical activity) to be obtained, strategies to increase accelerometer wear-time would need to be implemented in a full trial. Preliminary data showed no evidence of intervention effectiveness and low intervention attendance rates. However, there was high implementation fidelity by facilitators, and the intervention was seen as acceptable and deliverable. A revised intervention building on successful intervention elements and incorporating strategies to improve attendance should therefore be considered.
